# Galangin ameliorates cardiac remodeling via the MEK1/2–ERK1/2 and PI3K–AKT pathways

**DOI:** 10.1002/jcp.28216

**Published:** 2019-02-11

**Authors:** Hui‐Bo Wang, Si‐Hui Huang, Man Xu, Jun Yang, Jian Yang, Ming‐Xin Liu, Chun‐Xia Wan, Hai‐Han Liao, Di Fan, Qi‐Zhu Tang

**Affiliations:** ^1^ Department of Cardiology Hubei Key Laboratory of Cardiology Cardiovascular Research Institute of Wuhan University Renmin Hospital of Wuhan University Wuhan People's Republic of China; ^2^ Department of Cardiology The First College of Clinical Medical Science China Three Gorges University Institute of Cardiovascular Diseases Yichang People's Republic of China

**Keywords:** AKT, cardiac remodeling, ERK1/2, fibrosis, galangin, GATA4, hypertrophy

## Abstract

Cardiac remodeling is associated with inflammation and apoptosis. Galangin, as a natural flavonol, has the potent function of regulating inflammation and apoptosis, which are factors related to cardiac remodeling. Beginning 3 days after aortic banding (AB) or Sham surgery, mice were treated with galangin for 4 weeks. Cardiac remodeling was assessed according to echocardiographic parameters, histological analyses, and hypertrophy and fibrosis markers. Our results showed that galangin administration attenuated cardiac hypertrophy, dysfunction, and fibrosis response in AB mice and angiotensin II‐treated H9c2 cells. The inhibitory action of galangin in cardiac remodeling was mediated by MEK1/2–extracellular‐regulated protein kinases 1/2 (ERK1/2)–GATA4 and phosphoinositide 3‐kinase (PI3K)–protein kinase B (AKT)–glycogen synthase kinase 3β (GSK3β) activation. Furthermore, we found that galangin inhibited inflammatory response and apoptosis. Our findings suggest that galangin protects against cardiac remodeling through decreasing inflammatory responses and apoptosis, which are associated with inhibition of the MEK1/2–ERK1/2–GATA4 and PI3K–AKT–GSK3β signals.

## 1 INTRODUCTION

Heart failure (HF) is the common end‐stage of all types of cardiovascular diseases. HF remains a major global clinical and public health challenge with high hospitalization and mortality (Fan et al., 2017). Cardiac remodeling is an adaptive process that is activated in response to various biomechanical stressors, such as hemodynamic overload, ischemia, hypoxia, and diabetes. Cardiac remodeling is characterized by a series of structural and functional alterations, including changes in left ventricle (LV) geometry, cardiomyocyte hypertrophy, and interstitial fibrosis (Ma et al., 2018). However, under conditions of sustained stress, these changes ultimately led to the initiation and progression of adverse effects, eventually leading to HF. The mechanistic processes of HF and cardiac remodeling remain to be fully elucidated (Wu et al., 2018). The effects of biomechanical stress stimuli, such as pressure overload and various humoral factors, for example, angiotensin II [Ang II] and transforming growth factor‐β [TGFβ], on cardiac intracellular signaling cascades are crucial for elucidating the molecular mechanism underlying cardiac remodeling. Therefore, clarifying the underlying mechanism of cardiac remodeling and pharmacological interventions targeting these molecular changes may lead to new therapeutic strategies for delaying or reversing the progress of HF.

As a member of mitogen‐activated protein kinases (MAPKs), extracellular‐regulated protein kinases 1/2 (ERK1/2) is considered the essential regulator of the hypertrophic response. ERK1/2 activation via phosphorylation of serine/threonine residues is triggered by MAPK/ERK kinase 1/2 (MEK1/2; Cai et al., 2009). Protein kinase B (AKT) is a serine/threonine kinase involved in the regulation of cardiac growth, proliferation, and migration. Activated‐phosphoinositide 3‐kinase (PI3K) promotes the activation of AKT, directly contributing to the process of cardiac remodeling (J. Li et al., 2017). Overexpression of activated AKT or ERK1/2 contributes to cardiac remodeling, whereas AKT or ERK1/2 knockout mice exhibit inhibition of the intracellular transduction cascades associated with cardiac remodeling.

In recent years, many studies have focused on the beneficial effects of flavonoids for alleviating cardiac remodeling. Flavonoids typically have less toxicity and side effects than chemically synthesized drugs (Zhang et al., 2016). Galangin (3,5,7‐trihydroxyflavone or 3,5,7‐trihydroxy‐2‐phenylchromen‐4‐one) is a natural flavonol, which is abundant in honey and *Alpinia officinarum* (a plant that has been used as a kind of herbal medicine for multiple ailments in Asia for centuries [Huang et al., 2017]). A series of related studies have demonstrated that galangin possesses several biological activities, including antioxidant (Aloud, Veeramani, Govindasamy, Alsaif, & Al‐Numair, 2018), anti‐inflammatory and antiapoptosis (Huang et al., 2017), anticancer (Y. Wang et al., 2017), and antifibrotic activities (X. Wang et al., 2013). However, the effects of galangin on cardiac remodeling and the potential signaling mechanisms have not yet been elucidated. The aim of this study is to determine whether galangin can attenuate cardiac remodeling induced by pressure overload in vivo and in Ang II cultured‐H9c2 cells in vitro, as well as to identify the mechanisms involved in these effects.

## 2 MATERIALS AND METHODS

### 2.1 Materials

Galangin was purchased from Winberb Medical S&T Development (Shanghai, China) with a purity of 99.24% as determined by high‐performance liquid chromatography analysis. Ang II was purchased from Sigma‐Aldrich (St. Louis, MO). The antibodies used to recognize total and phosphorylated MEK1/2, ERK1/2, PI3K, AKT, glycogen synthase kinase 3β (GSK3β), Smad2, as well as TGFβ, Bax, Bcl2, and glyceraldehyde‐3‐phosphate dehydrogenase (GAPDH) were purchased from Cell Signaling Technology (Danvers, MA). Anti‐GATA4 and anti‐phospho‐GATA4 were obtained from Abcam (Cambridge, UK). 4′,6‐Diamidino‐2‐phenylindole (DAPI; S36939) was purchased from Invitrogen. Protein assay kits were obtained from Pierce (23225; Pierce).

### 2.2 Animals and treatments

Eight‐week‐old male C57/BL6 mice (23.5–25.5 g) were purchased from the Institute of Laboratory Animal Science, CAMS&PUMC (Beijing, China). The animals were housed in a specific pathogen‐free barrier with controlled temperature and humidity. The experimental procedures were approved by the Institutional Guidelines of the Animal Care and Use Committee of Renmin Hospital, which is compliant with the Guide for the Care and Use of Laboratory Animals (NIH Publication No. 85‐23, revised 2011). The diet was based on commonly used diets. Aortic banding (AB) and the corresponding Sham operation were performed in 60 and 30 mice, respectively, after acclimatizing them to the laboratory environment for 1 week as described in previous articles (Ma et al., 2018). The AB operation and data analyses were performed in a blinded fashion for all groups. Three days after the AB or Sham operation, the animals were treated with the same volume of vehicle (0.5% carboxymethyl cellulose solution) or galangin (5, 25, and 50 mg/kg body weight/day, suspended in 0.5% carboxymethyl cellulose solution) daily for 4 weeks after surgery. At the end of the treatment, the mice were killed by cervical dislocation, and the hearts were dissected and weighed to compare heart weight/body weight (HW/BW, mg/g), lung weight/body weight (LW/BW, mg/g), and heart weight/tibia length (HW/TL, mg/mm) among the six groups.

### 2.3 Echocardiography

Mice were anesthetized by 1.5% isoflurane. Echocardiography was used to detect cardiac function in each group of mice, which was performed with a MyLab 30CV system (Biosound Esaote, Inc.) with a 10‐MHz phased array transducer. Two‐dimensional guided M‐mode echocardiographic images were obtained at the papillary muscle. End‐diastole and end‐systole were defined as the phases in which the largest and smallest areas of the LV were obtained, respectively. The LV end‐systolic diameter (LVED), LV end‐diastolic diameter (LVEDd), and posterior wall thickness (PWT) were measured via LV M‐mode tracing with a sweep speed of 50 mm/s. LV‐ejection fraction (EF) and fractional shortening (FS) were calculated by the LVEDs and LVEDd values.

### 2.4 Histological analysis

The arrested hearts were placed in 10% potassium chloride solution immediately to ensure that they were stopped in diastole, and fixed with 10% formalin. The hearts were cut transversely to visualize the LVs and right ventricles and embedded in paraffin. Sections of each heart (5‐μm thick) were prepared and stained with hematoxylin and eosin for the evaluation of cross‐sectional areas (CSAs) or 0.1% Picro‐Sirius red (PSR) for the evaluation of collagen deposition. Subsequently, the slides were visualized by light microscopy (ECLIPSE 80i; Nikon, Japan). Image‐Pro Plus version 6.00 (IPP6.0) was used to view the myocardial cross‐sectional area, to trace a single myocyte (150–200 myocytes in each group), and to measure collagen content.

### 2.5 Quantitative real‐time reverse transcription‐polymerase chain reaction

For real‐time polymerase chain reaction (RT‐PCR), total RNA was extracted from LV tissue using TRIzol (15596‐026; Invitrogen). The concentration and purity of the extracted RNA were spectrophotometrically estimated by the A260/A280 and A260/230 ratios via an ultraviolet spectrophotometer (NanoDrop2000; Thermo Fisher Scientific). RNA (2 μg of each sample) was reverse‐transcribed into complementary DNA (cDNA) using oligo (DT) primers and a Transcriptor First Strand cDNA Synthesis Kit (No. 04896866001; Roche, Germany). PCR amplifications were performed using LightCycler 480 SYBR Green 1 Master Mix (No. 04707516001; Roche, Germany) according to the manufacturer's instructions. All of the primer information is provided in Table 1. The messenger RNA (mRNA) levels were analyzed with the $2^{-\Delta \Delta C_{t}}$ method and normalized against GAPDH gene expression.

**Table 1:** The primer sequences for RT‐PCR

Targets	Forward	Reverse
Mice‐GAPDH	TCATCAACGGGAAGCCCATC	CTCGTGGTTCACACCCATCA
Mice‐ANP	ACCTGCTAGACCACCTGGAG	CCTTGGCTGTTATCTTCGGTACCGG
Mice‐BNP	GAGGTCACTCCTATCCTCTGG	GCCATTTCCTCCGACTTTTCTC
Mice‐β‐MHC	CCGAGTCCCAGGTCAACAA	CTTCACGGGCACCCTTGGA
Mice‐IL‐1β	CCGTGGACCTTCCAGGATGA	GGGAACGTCACACACCAGCA
Mice‐IL‐6	AGTTGCCTTCTTGGGACTGA	TCCACGATTTCCCAGAGAAC
Mice‐collagen1a	AGGCTTCAGTGGTTTGGATG	CACCAACAGCACCATCGTTA
Mice‐collagen III	AAGGCTGCAAGATGGATGCT	GTGCTTACGTGGGACAGTCA
Mice‐αSMA	GTCCCAGACATCAGGGAGTAA	TCGGATACTTCAGCGTCAGGA
Rat‐GAPDH	GACATGCCGCCTGGAGAAAC	AGCCCAGGATGCCCTTTAGT
Rat‐ANP	AAAGCAAACTGAGGGCTCTGCTCG	TTCGGTACCGGAAGCTGTTGCA
Rat‐BNP	CAGCAGCTTCTGCATCGTGGAT	TTCCTTAATCTGTCGCCGCTGG
Rat‐β‐MHC	TCTGGACAGCTCCCCATTCT	CAAGGCTAACCTGGAGAAGATG

*Note*. GAPDH: glyceraldehyde‐3‐phosphate dehydrogenase; IL‐6: interleukin 6; RT‐PCR: real‐time polymerase chain reaction; β‐MHC: β‐myosin heavy chain.

### 2.6 Western blot

For western blot, frozen cardiac tissue and cultured cells were lysed in radioimmunoprecipitation assay lysis buffer, and an amount of protein was assessed using the BCA Protein Assay Kit (Thermo Fisher Scientific). The protein concentration in all samples was normalized before western blot. The protein lysates (50 μg) were separated by 8, 10 or 12% sodium dodecyl sulfate‐polyacrylamide gel electrophoresis, and proteins were subsequently transferred to polyvinylidene fluoride membranes (FL00010; EMD Millipore) as previously described. The membrane was blocked with 5% nonfat milk (Tris‐buffered saline with 0.1% Tween 20) at room temperature for 1 hr, and then incubated with primary antibodies overnight at 4°C on a rocking platform. The next day, after washing three times with Tris‐buffered saline with 0.1% Tween 20, the blots were incubated with 1:1000 dilution of secondary antibodies for 1 hr. The scanning and quantification of protein blots were performed with an infrared Li‐Cor scanner (Odyssey, LI‐COR), and the protein expression levels were normalized to GAPDH protein.

### 2.7 Cell culture

H9c2 cells were obtained from the Cell Bank of the Chinese Academy of Sciences (Shanghai, China) and cultured in Dulbecco's modified Eagle's medium (C11995; GIBCO) containing 10% fetal bovine serum (C10099; GIBCO) and placed in a humidified incubator (SANYO 18 M, Japan) containing 5% CO_2_ at 37°C. H9c2 cells were seeded in six‐well plates (1 × 10^6^ cells/well) for western blot analysis and PCR detection, or seeded in 24‐well plates (3 × 10^3^ cells/well) for immunofluorescence staining and terminal deoxynucleotide transferase‐mediated dUTP nick end‐labeling (TUNEL) staining. The H9c2 cells were averaged and divided into four groups: the control group (Con), the Ang II (1 μM; Sigma) treatment group, the Gal (5, 25, 50 μM) group, and the Ang II + Gal (5, 25, 50 μM) group. After treatment for 24 hr, cells from the six‐well plates and 24‐well plates were harvested for PCR, western blot analysis, and immunofluorescence staining analyses.

### 2.8 Cell viability assay

Cell viability was evaluated using the Cell Counting Kit‐8 (CCK8) assay (GB707; Dojindo, Japan), in accordance with the manufacturer's instructions. Following galangin treatment with a gradient concentration (six repeated wells in each concentration) for 24 hr, 10 μl of a CCK8 solution was added to each well in a 96‐well plate and the absorbance was measured at 450 nm by an ELISA reader (SynergyHT; Bio Tek) after 4 hr of incubation. The effect of different concentrations of galangin on cell viability was expressed as the percentage of cell viability compared with the vehicle group, which was set at 100%.

### 2.9 Immunofluorescence staining

To identify the H9c2 cells and assess the levels of H9c2 cells hypertrophy, the cells were characterized using immunofluorescence staining for the marker α‐actinin (05–384; Millipore). For this experiment, the cells were washed for 3 × 5 min with phosphate‐buffered saline (PBS), fixed with 4% paraformaldehyde for 15 min, permeabilized with 0.2% Triton X‐100 (0694; Amresco) for 5 min in PBS, and blocked with 8% goat serum in PBS for 1 hr. The cells were then stained with anti‐α‐actinin antibody at a dilution of 1:100 with 1% goat serum overnight at 4°C. The coverslips were incubated with a secondary antibody (goat antimouse IgG Alexa Fluor 488) at 37°C for 1 hr. The cells were washed five times with warm PBS and then mounted onto glass slides with DAPI. Immunofluorescence images were taken on a fluorescence microscope (OLYMPUS DX51; Japan) and the CSAs were measured using IPP6.0. We traced the outline of 100‐H9c2 cells in each group.

### 2.10 Assessment of apoptosis

Apoptosis was assessed using TUNEL staining according to the instructions of the Apoptosis Detection Kit (S7111; Chemicon). Briefly, cells on coverslips were fixed in 4% paraformaldehyde in PBS and stained with TUNEL reagents and DAPI. The cell apoptotic index was calculated as the ratio of the number of apoptotic nuclei to the total number of nuclei.

### 2.11 Statistical analysis

All values are presented as the means ± standard error of the means and were analyzed by GraphPad Prism 5.0 software (GraphPad Software). One‐way analysis of variance followed by post hoc Tukey's test was used for data analysis. Comparisons between two groups were performed by unpaired, two‐tailed Student's *t* tests. A value of *p* < 0.05 was considered statistically significant.

## 3 RESULTS

### 3.1 Galangin attenuated cardiac hypertrophy and improved cardiac function in vivo

**Figure 1:**
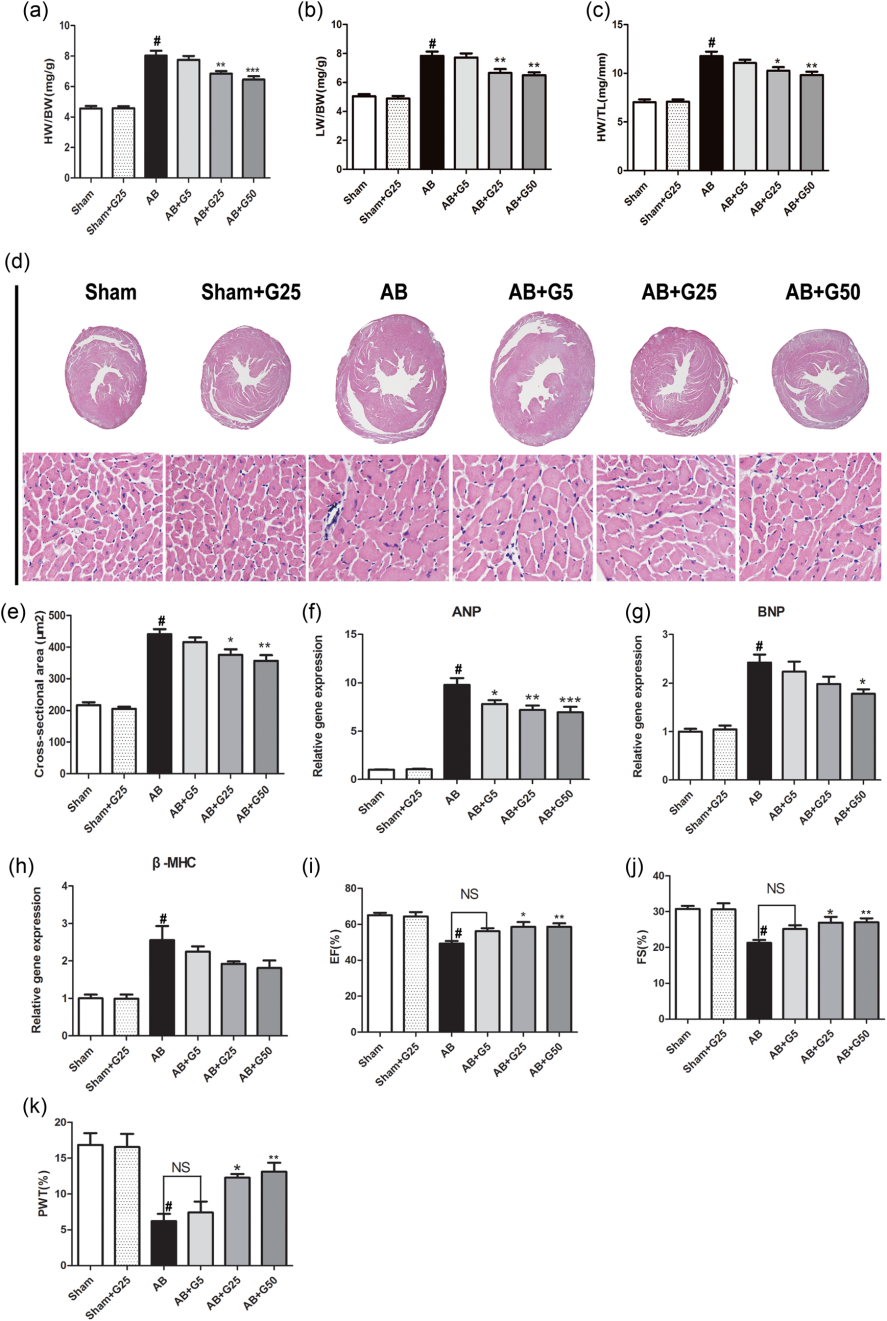
Galangin attenuated cardiac hypertrophy and improved cardiac function induced by pressure overload in vivo. (a–c) Statistical results of HW/BW ratio, LW/BW ratio, and HW/TL ratio at 4 weeks after AB surgery (*n* = 10). (d) HE staining of Sham and AB mice at 4 weeks after surgery in treatment by vehicle and Galangin mice (*n* = 6); (e) Statistical results for the cross‐sectional area (*n* = 6). (f–h) The mRNA levels of hypertrophic markers (*n* = 6). (i–k) Echocardiographic parameters in galangin‐treated mice (*n* = 8). ^#^ *p* < 0.05 versus Sham group; *p < 0.05 or **p < 0.01, versus AB group. AB: aortic banding; HE: haematoxylin and eosin; HW/BW: heart weight/body weight; HW/TL: heart weight/tibia length; LW/BW: lung weight/body weight; mRNA: messenger RNA [Color figure can be viewed at wileyonlinelibrary.com]

To explore the effects of galangin on cardiac hypertrophy in a murine model, we established an AB‐mediated pressure overload model. Hypertrophy measured by HW/BW and HW/TL, hypertrophic genes (ANP, BNP, and β‐MHC), CSA, and echocardiography. To verify the toxicity and side effects of galangin on the heart, liver, and kidney, we used a maximum dose of 50 mg/Kg/Day in this study (shown in supplementary data 1). The dose of 50 mg/Kg/Day galangin has almost no toxic and side effects on the heart, liver, lung, and kidney. All AB mice demonstrated a marked increase in heart weight and lung weight as compared with the Sham control group after 4 weeks postsurgery. In contrast, middle‐dose (25 mg/kg/day) and high‐dose (50 mg/kg/day) galangin treatment of the AB mice significantly decreased hypertrophic growth as measured by HW/BW, LW/BW, and HW/TL (Figure 1a–c). No significant differences were observed in the AB mice treated with low dose (5 mg/kg/day) galangin treatment or vehicle, as well as Sham‐operated mice treated with galangin or vehicle. Consistently, the expression of hypertrophic genes (ANP, BNP, and β‐MHC) and CSA were more or less decreased in the middle‐dose and high‐dose galangin‐treated mice after AB (Figure 1d–h). There were no significant changes in these indices in the Sham‐surgery group after treatment with vehicle or galangin. The effect of galangin on ventricular dysfunction after AB was further confirmed by increasing the percentage of LV–EF, LV–FS, and PWT (the percentage change of PWT between diastole and systole was measured; Figure 1i–k). These results suggest that the middle‐dose (25 mg/kg/day) and high‐dose (50 mg/kg/day) galangin treatment could improve cardiac function and suppress cardiac hypertrophy after TAC surgery, but there was no significant difference in the effects of low dose (5 mg/kg/day) galangin on hypertrophy and cardiac function.

### 3.2 Galangin attenuate cardiac fibrosis in vivo

**Figure 2:**
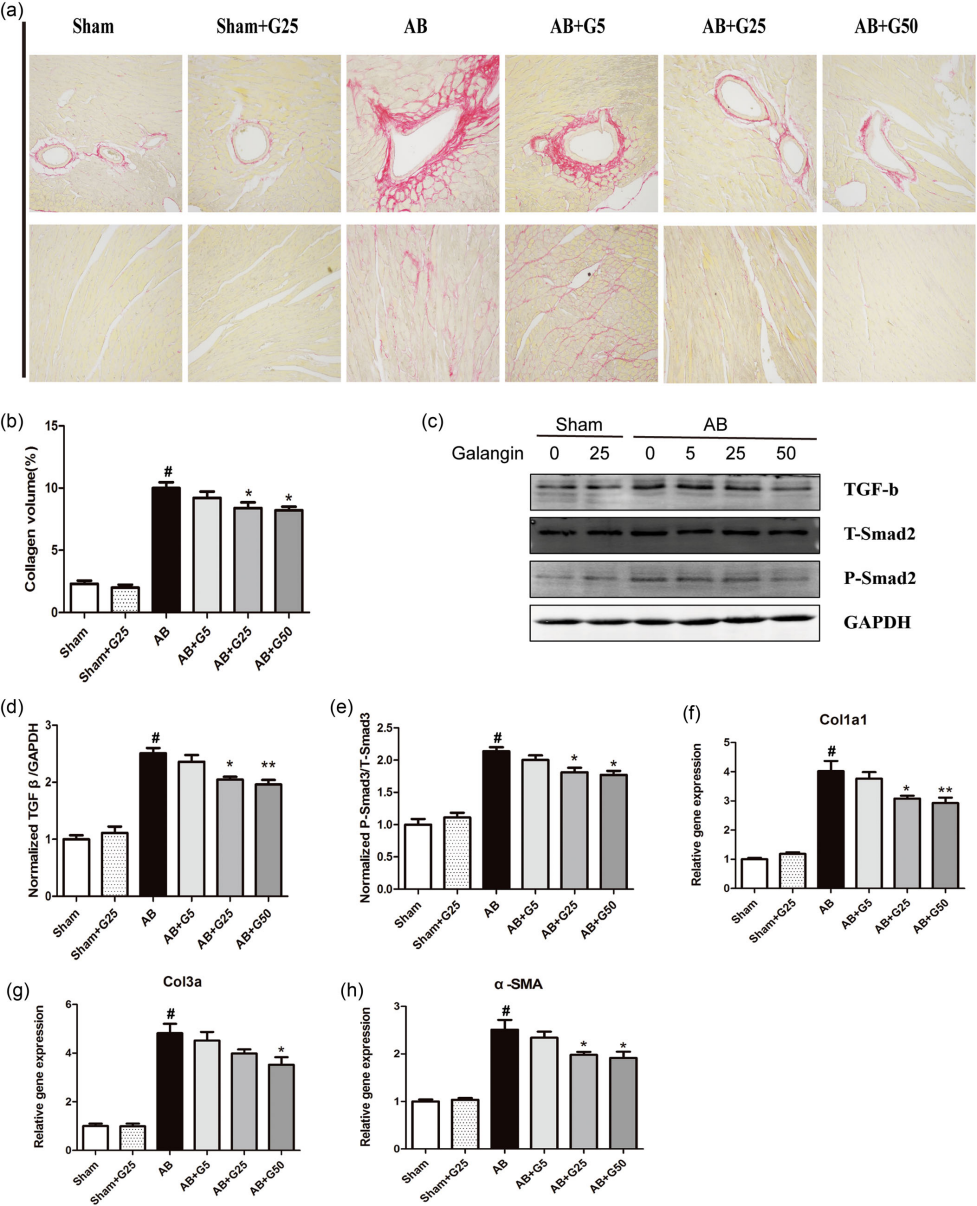
Galangin attenuated cardiac fibrosis induced by pressure overload in vivo. (a,b) Representative PSR staining on histological sections of the LV and the statistical results (6 = 5). (c–e) Representative blots of TGF‐β1, Smad2 phosphorylation, and Smad2 from indicated groups (*n* = 6). (d) Real‐time PCR analyses of fibrosis‐related genes (*n* = 6). ^#^ *p* < 0.05 versus Sham group; *p < 0.05 or **p < 0.01, versus AB group. AB: aortic banding; LV: left ventricle; PCR: polymerase chain reaction; PSR, Picro‐Sirius red; TGF‐β1: transforming growth factor β1; α‐SMA: α‐smooth muscle actin [Color figure can be viewed at wileyonlinelibrary.com]

The extent of cardiac interstitial fibrosis was determined, as indicated by increased‐LV collagen volume (evaluated by PSR) and fibrotic markers. After 4 weeks of AB, dramatic cardiac perivascular and interstitial fibrosis were observed in all AB surgery mice, but the extent of fibrosis was markedly reduced in middle‐dose (25 mg/kg/day) and high‐dose (50 mg/kg/day) galangin‐treated mice (Figure 2a,b). To examine the potential molecular mechanisms of galangin in collagen synthesis, we assessed the effect of galangin on TGF‐β1/Smad cascade activation. The increased levels of TGF‐β1 and phosphorylated Smad2 were inhibited in middle‐dose (25 mg/kg/day) and high‐dose (50 mg/kg/day) galangin‐treated mice after AB surgery (Figure 2c–e). The subsequent analysis of mRNA collagen I, collagen III, and α‐smooth muscle actin was also downregulated by middle‐dose and high‐dose galangin‐treated mice after AB surgery (Figure 2f–h). These results indicate that mice treated with middle‐dose and high‐dose galangin‐treated mice showed a limitation in fibrotic response induced by AB surgery.

### 3.3 Galangin attenuate Ang II‐induced H9c2 cells hypertrophy in vitro

**Figure 3:**
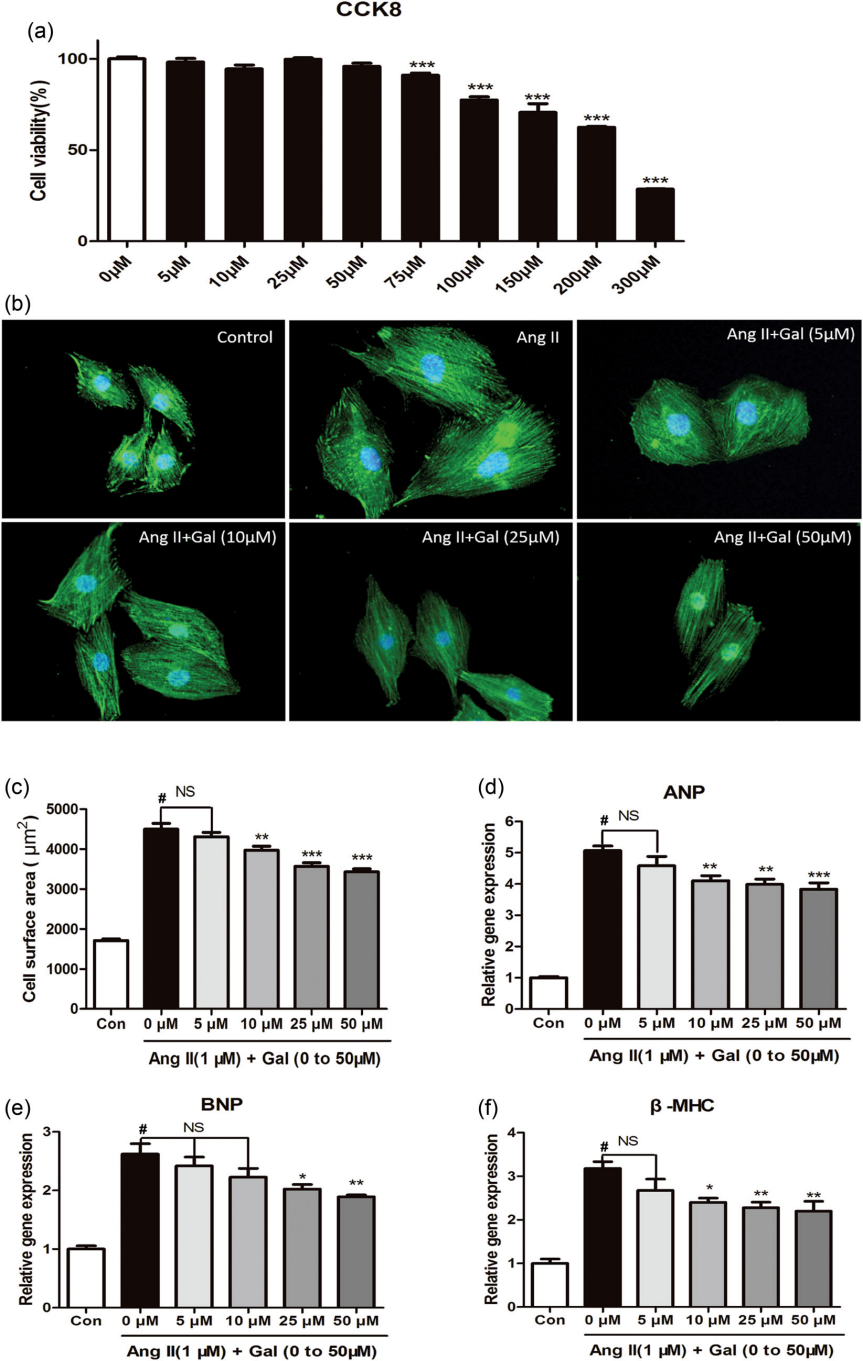
Galangin suppresses Ang II‐induced H9c2 cardiomyocyte hypertrophy. (a) Cell viability was accessed by the Cell Counting Kit‐8 assay. Cell Counting Kit‐8 assay was used to detect the cell viability of H9c2 cells in different concentrations (0, 5, 10, 25, 50, 75, 100, 150, 200, and 300 μM) of galangin (*n* = 6). (b, c) Immunofluorescence staining of α‐actinin and the cell surface area of H9c2 cells in the indicated groups (*n* = 6 samples, and 100 + cells per group). (d, f) H9c2 cells were stimulated with 1 μM Ang II and treated with galangin (0, 5, 10, 25, and 50 mM). The mRNA levels of ANP, BNP, and β‐MHC in H9c2 cells in each group (*n* = 6). ^#^ *p* < 0.05 versus Sham group or control group; *p < 0.05 or **p < 0.01, versus AB or Ang II group. AB: aortic banding; Ang II: angiotensin II; CCK8: Cell Counting Kit‐8; mRNA: messenger RNA [Color figure can be viewed at wileyonlinelibrary.com]

To rule out the possibility of galangin in H9c2 cells cytotoxicity, we determined cell viability by CCK‐8 assay. Our data showed that galangin was noncytotoxic for H9c2 cells when the concentration was below 75 μM for 24 hr (Figure 3a). To further confirm the effect of galangin on cardiac hypertrophy, we established an in vitro model with 1 μM Ang II in cultured‐H9c2 cells. After stimulation with galangin (0, 5, 10, 25, 50 μM) with or without Ang II for 24 hr, H9c2 cells were characterized for cardiac α‐actinin by immunofluorescence, to assess H9c2 cells cell surface area. After stimulation with Ang II, vehicle‐treated H9c2 cells showed increased cell surface area compared with those induced by galangin (10, 25, 50 μM) with Ang II for 24 hr (Figure 3b,c). H9c2 cells hypertrophy was also assessed by RT‐PCR; galangin (10, 25, and 50 μM) markedly decreased the level of ANP, BNP, and β‐MHC mRNA induced by Ang II, most significantly in H9c2 cells treated with 50 μM galangin (Figure 3d,f). These findings indicated that galangin attenuated H9c2 cells hypertrophy in vitro. On the basis of these results, the concentration of 50 μM galangin was selected for further investigations.

### 3.4 Galangin attenuate inhibited cardiomyocyte apoptosis

**Figure 4:**
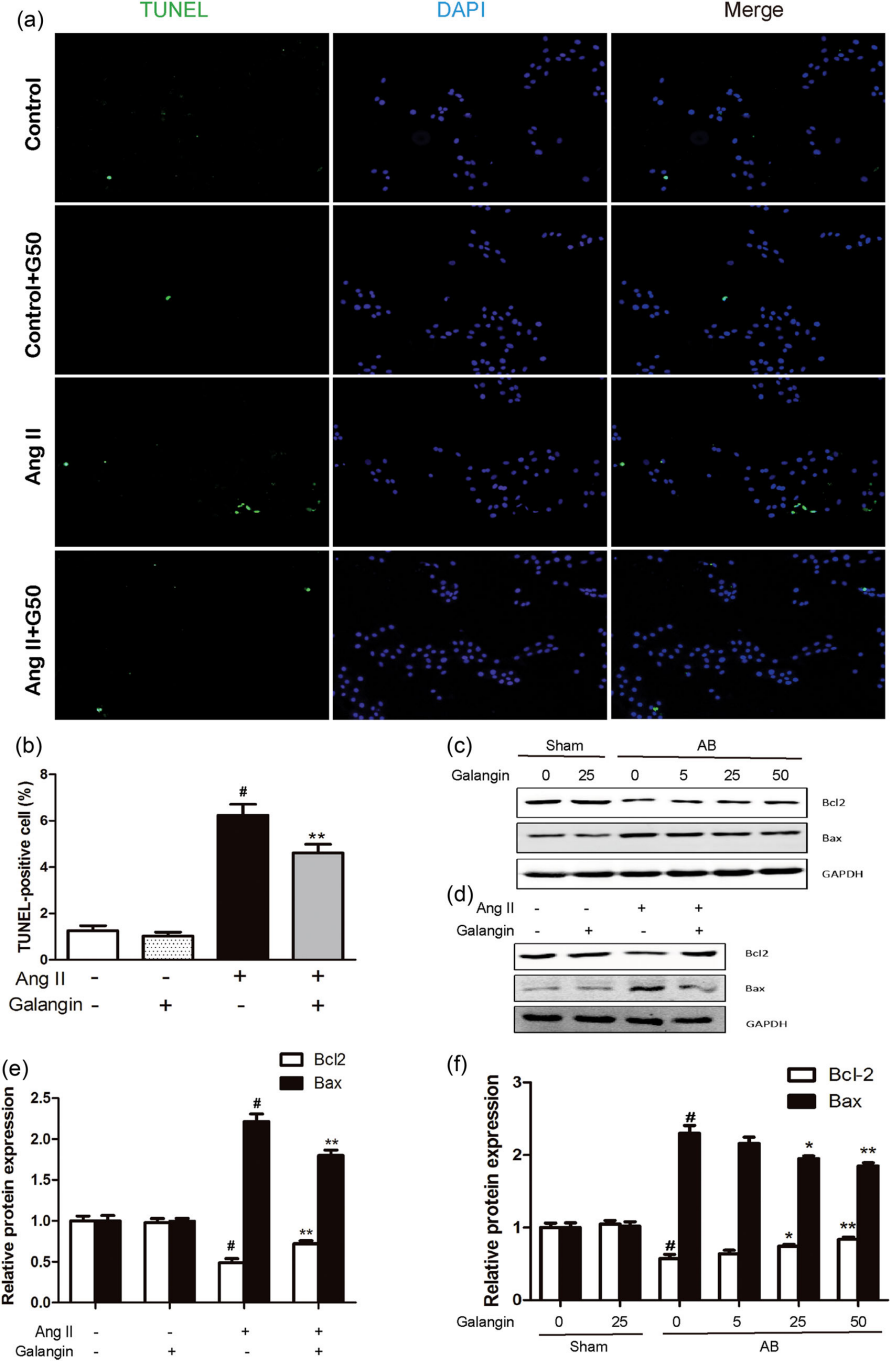
Galangin protected against cardiomyocyte apoptosis. (a) TUNEL staining exhibited the cellular apoptosis in vitro. (b) Counting of the TUNEL‐positive nuclear. (c–f) Representative western blot demonstrating Bcl2 and Bax protein expressions with the GAPDH in vitro and in vivo. *^#^p* < 0.05 versus Sham group or control group. *p < 0.05 or **p < 0.01, versus AB or Ang II group. AB: aortic banding; Ang II: angiotensin II; GAPDH: glyceraldehyde‐3‐phosphate dehydrogenase; TUNEL: transferase‐mediated dUTP nick end‐labeling [Color figure can be viewed at wileyonlinelibrary.com]

TUNEL assay was performed to assess the Ang II‐induced‐cell apoptosis in cultured‐H9c2 cells. Only 1.255 ± 0.221% and 1.021 ± 0.178% TUNEL‐positive nuclei were, respectively, detected in the Con and Con + Gal group cells. A significantly increased percentage of TUNEL‐positive nuclei was observed in cells incubated with Ang II (6.240 ± 0.471%; *p* < 0.001 vs. the control group); however, galangin treatment significantly reduced the percentage of TUNEL‐positive cells (4.610 ± 0.376%; *p* < 0.01 vs. the Ang II‐only group; Figure 4a,b). We subsequently investigated the expression of the apoptosis‐related protein Bax and Bcl2 in vitro and in vivo. We found that Ang II treatment or AB surgery significantly upregulated proapoptotic protein Bax, and downregulated antiapoptotic protein Bcl2; however, galangin increased Bcl2 expression and decreased Bax expression (Figure 4c–e). These data indicated that galangin protected H9c2 cells from Ang II or AB surgery‐induced apoptosis.

### 3.5 Galangin attenuate AB or Ang II‐mediated inflammatory responses in vivo and in vitro

**Figure 5:**
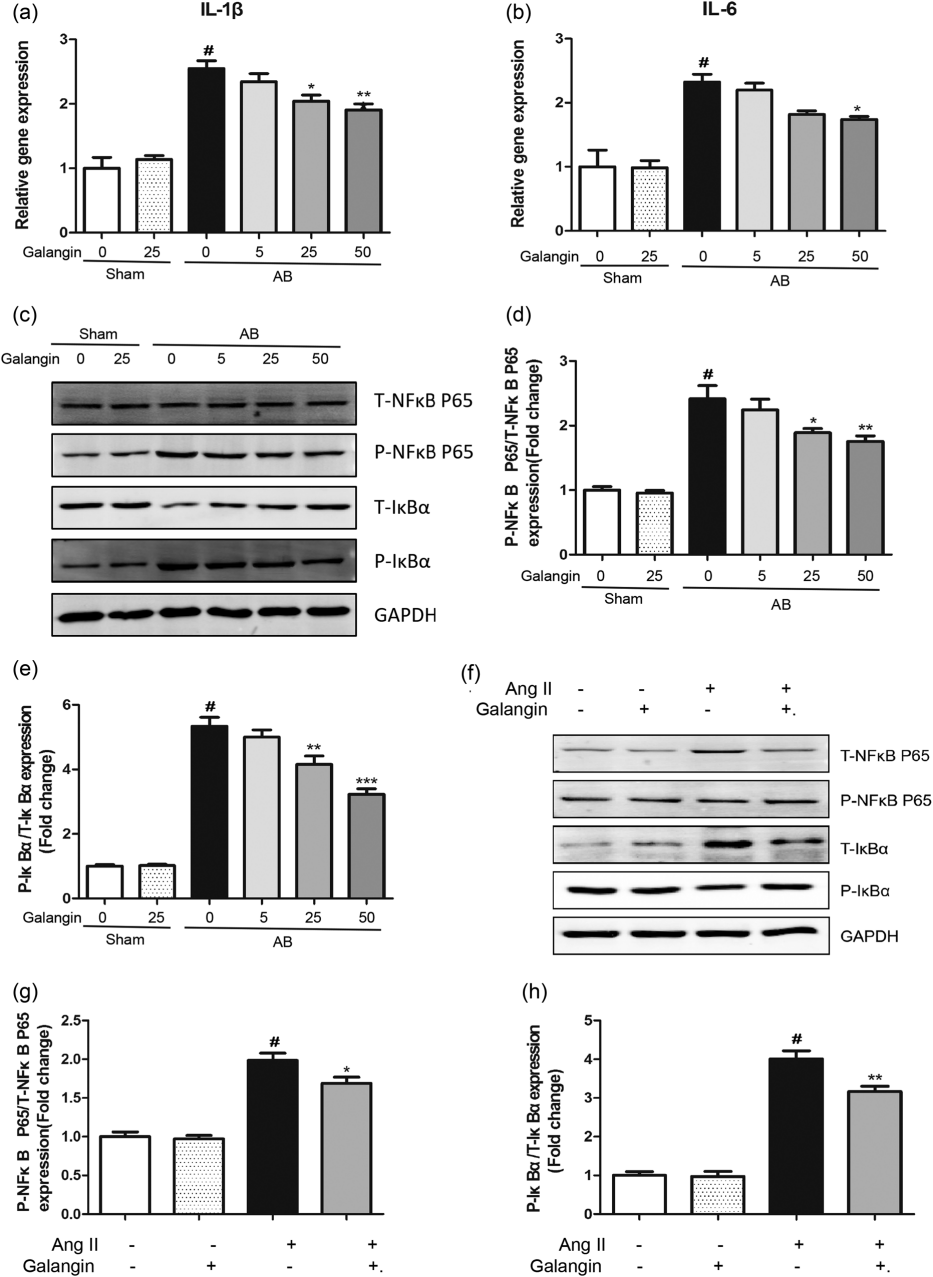
Galangin protected against cardiomyocyte inflammatory responses in vivo and in vitro. (a, b) The mRNA expression of inflammatory cytokines IL‐1 cytok‐6 in mouse heart. (c–h) Representative blots and quantification of P‐NFκB‐P65/T‐NFκB‐P65 and P‐IκBα/T‐IκBακ in vivo and in vitro. ^#^ *p* < 0.05 versus Sham group or control group. *p < 0.05 or **p < 0.01, versus AB or Ang II group. AB: aortic banding; Ang II: angiotensin II; mRNA: messenger RNA

To detect the level of inflammatory responses, the levels of inflammatory cytokines (interleukin [IL]‐1β and IL‐6) were detected by the RT‐PCR and the NF‐κB pathway (P‐NF‐κB P65/T‐NF‐ΚB P65 and P‐IκBα/T‐IκBα) by western blot. We found increased mRNA and protein expression levels of IL‐1β, IL‐6, P‐NF‐κB P65/T‐NF‐ΚB P65 ratio, P‐IκBα/T‐IκBα ratio at 4 weeks postAB surgery in vivo and postAng II stimulus in vitro, whereas the expression of levels of IL‐1β, IL‐6, P‐NF‐κB P65/T‐NF‐ΚB P65 ratio, P‐IκBα/T‐IκBα ratio were dramatically decreased in the middle‐dose (25 mg/kg/day) and high‐dose (50 mg/kg/day) galangin‐treated mice in vivo and 50 μM galangin stimulus in vitro (Figure 5a–h).

### 3.6 Effects of galangin on PI3K–Akt–GSK3β signaling in vitro and in vivo

**Figure 6:**
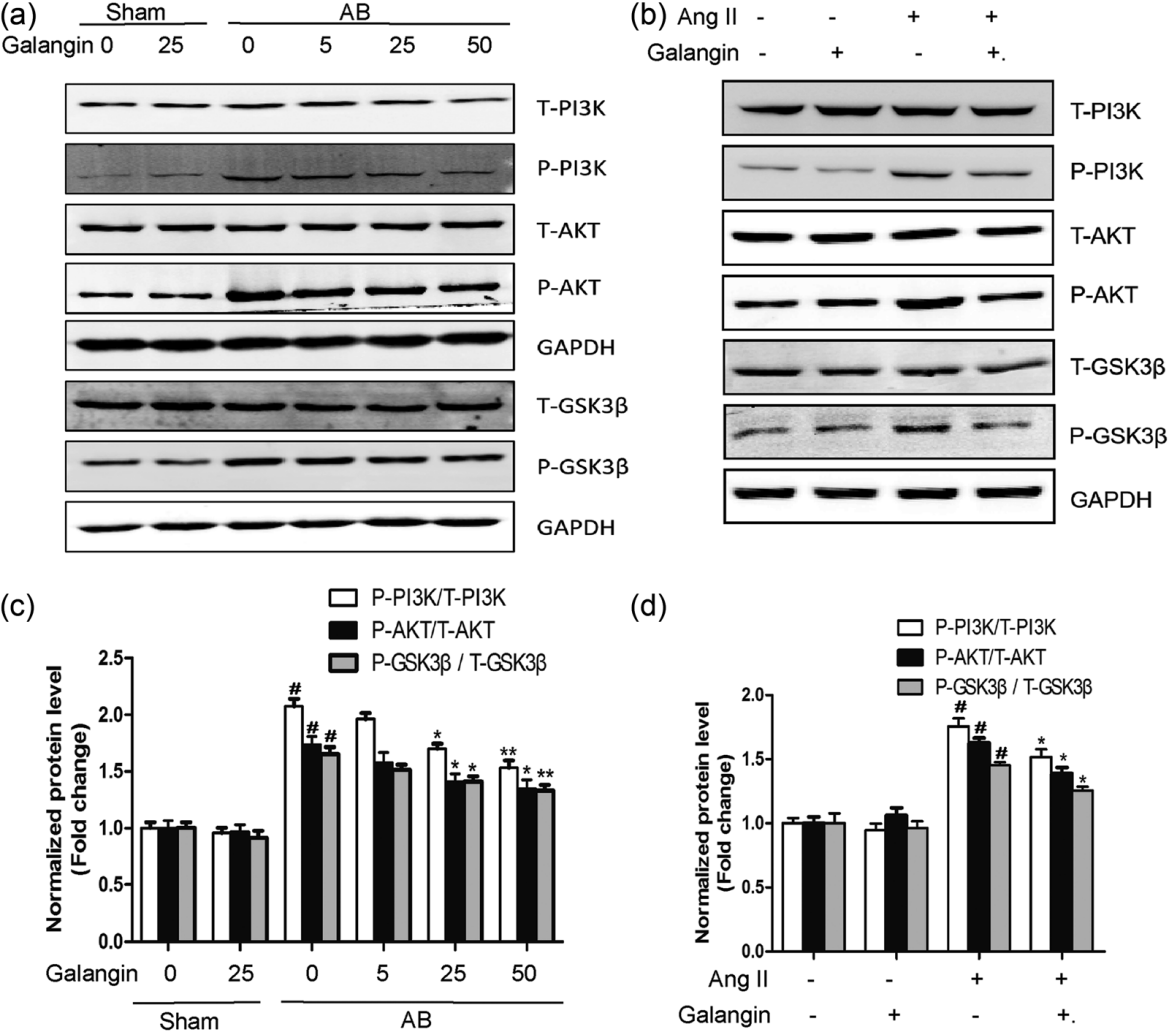
Effect of galangin on the PI3K–AKT‐GSK3β signaling pathway. (a, b) Representative western blots for total and phosphorylation of PI3K, AKT, GSK3β. (c, d) Quantitative results of western blot (*n* = 5/ group). ^#^ *p* < 0.05 versus Sham group or control group. *p < 0.05 or **p < 0.01, versus AB or Ang II group. AB: aortic banding; AKT: protein kinase B; Ang II: angiotensin II; GSK3β: glycogen synthase kinase 3β; PI3K: phosphoinositide 3‐kinase

To investigate the potential molecular mechanism of galangin on cardiac remodeling, we examined the effects of galangin on the PI3K–Akt–GSK3β signaling pathway. Our data showed that PI3K, Akt, GSK3β were significantly phosphorylated after treatment with Ang II in vitro or 4 weeks after AB surgery in vivo, and middle‐dose (25 mg/kg/day) and high‐dose (50 mg/kg/day) galangin treatment of mice in vivo and 50 μM galangin in vitro evidently blocked the activation of PI3K, Akt, and GSK3β (Figure 6a–d). The results showed that galangin significantly inhibits cardiac remodeling through inhibition of the PI3K–Akt–GSK3β signaling pathway.

### 3.7 Effects of galangin on MEK1/2–ERK1/2–GATA4 signaling in vitro and in vivo

**Figure 7:**
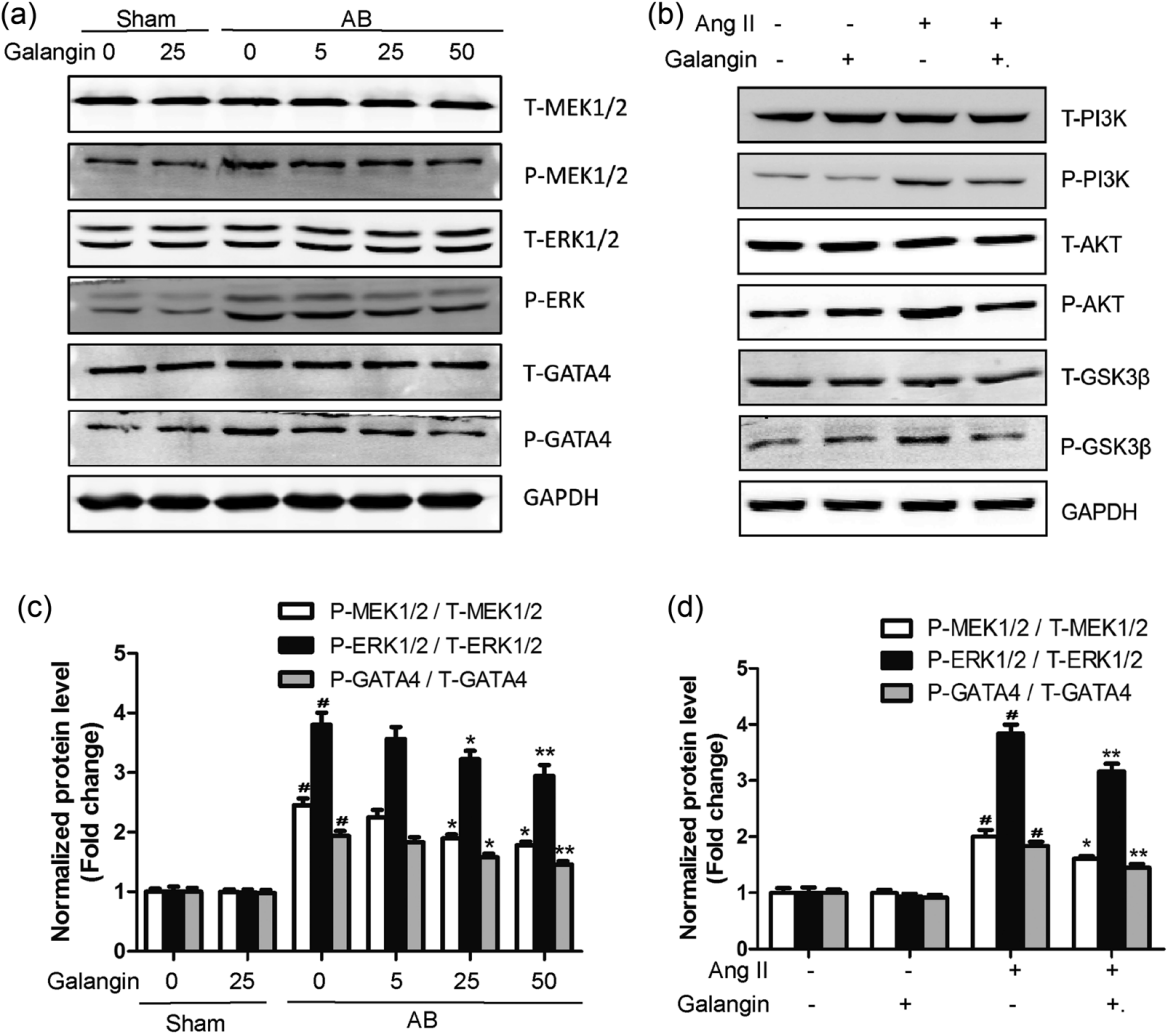
Effects of galangin on the MEK1/2–ERK1/2–GATA4 signaling pathway. (a, b) Representative western blots for total and phosphorylation of MEK1/2, ERK1/2, and GATA4. (c, d) Quantitative results of western blot (*n* = 5 per experimental group) ^#^ *p* < 0.05 versus the Sham group or the control group; *p< 0.05 or **p < 0.01, versus AB or Ang II group. AB: aortic banding; Ang II: angiotensin II; ERK1/2: extracellular‐regulated protein kinases 1/2

We found that the phosphorylated levels of MEK1/2, ERK1/2, and GATA4 were significantly increased in vehicle‐treated mice subjected to AB surgery in vivo and postAng II stimulus in vitro. However, the increased phosphorylated levels of MEK1/2, ERK1/2, and GATA4 were blocked in galangin (25, 50 mg/kg/day in vivo or 50 μM in vitro)‐treated hearts or H9c2 cells (Figure 7a–d). The results showed that galangin significantly inhibits cardiac remodeling through inhibition of the MEK1/2–ERK1/2–GATA4 signaling pathway.

## 4 DISCUSSION

**Figure 8:**
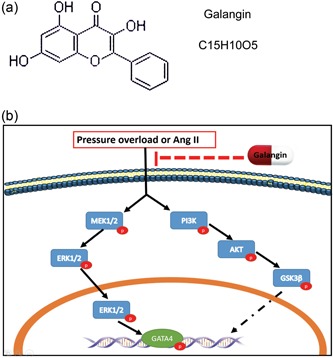
Galangin's molecular structure and proposed model of the effect of galangin on cardiac remodeling. (a) Representative galangin's molecular structure. (b) Representative proposed a model of the effect of galangin on cardiac remodeling. AKT: protein kinase B; Ang II: angiotensin II; ERK1/2: extracellular‐regulated protein kinases 1/2; PI3K: phosphoinositide 3‐kinase [Color figure can be viewed at wileyonlinelibrary.com]

In the present study, we examined the role of galangin in cardiac remodeling induced by AB‐mediated pressure overload in vivo and by Ang II in vitro. The results demonstrated that galangin significantly ameliorated cardiac function and inhibited cardiac hypertrophy and fibrosis by disruption of PI3K–Akt–GSK3β and MEK1/2–ERK1/2–GATA4 signaling after pressure overload in vivo and Ang II‐stimulation in vitro. According to the literature, this study proved, for the first time, that galangin plays an important protective role in regulating cardiac hypertrophy and fibrosis (Figure 8).

Cardiac remodeling is a pathophysiological process involving multiple complex factors in the process of HF. Its main manifestations are cardiomyocytes hypertrophy, changes in cardiac volume and geometry, myocardial fibrosis, and electrical remodeling (Wu et al., 2018). Multiple factors have been demonstrated to be involved in the development and progression of cardiac remodeling. Galangin is a member of the natural flavonol, which is abundant in honey and *A*. *officinarum* (a plant that has been used as a kind of herbal medicine for multiple ailments in Asia for centuries; Y. N. Liu et al., 2015). Galangin has attracted much attention for its potential anti‐inflammatory, antioxidant, apoptosis regulatory properties (Aloud, Chinnadurai, Govindasamy, Alsaif, & Al‐Numair, 2018; Lei et al., 2018; Y. N. Liu et al., 2015). Some studies have found that galangin, as a promising anti‐remodeling agent in asthma, may involve in the TGF–β1–ROS–MAPK pathway (Y. N. Liu et al., 2015). Meanwhile, galangin can inhibit CCl4‐mediated liver fibrosis in rats, its mechanism may be related to scavenging oxygen–free radicals, reducing lipid peroxidation, and inhibiting the activation and proliferation of hepatic stellate cells (X. Wang et al., 2013). However, the effects of galangin on cardiac remodeling and the potential signaling mechanisms have not yet been elucidated.

AKT mainly regulates cell survival and apoptosis. Recent studies have shown that PI3K–AKT signaling pathways play a considerable role in the process and pathogenesis of cardiac remodeling (J. Li et al., 2017). Previous studies from our laboratory or others have indicated that activated AKT directly phosphorylates and inactivates GSK3β, a series of processes that regulate the general protein translational machinery and participate in the development of pressure overload‐induced cardiac hypertrophy. Reducing the expression of Akt in the heart by gene knockout or RNA interference can inhibit the intracellular transduction cascade of cardiac hypertrophy (DeBosch et al., 2006; J. Li et al., 2017). Our team also demonstrated that inhibiting AKT/GSK3β attenuated pressure overload‐induced myocardial fibrosis and blocked cardiac fibroblasts activation and transformation in vitro (Yan et al., 2011). We observed that the phosphorylation levels of PI3K, AKT, and GSK3β are increased in the remodeling heart caused by pressure overload and in H9c2 cells under Ang II‐stimulation and that these effects are inhibited by galangin.

ERK1/2 (extracellular‐regulated protein kinases 1/2) is a class of serine/threonine protein kinases, which is a signal transduction protein that transmits mitogen signals. It is normally located in the cytoplasm and translocates to the nucleus when activated by phosphorylation, thus regulating the activity of transcription factors and producing corresponding cellular effects (Linke et al., 2014; Thei, Rocha‐Ferreira, Peebles, Raivich, & Hristova, 2018). As an important member of the MAPK signaling pathway, ERK1/2 can directly modify a series of transcription factors that promote cardiac gene expression and ultimately lead to cardiac hypertrophy (Ma et al., 2016). The activation of the ERK1/2 is triggered by MAPK1/2 (MEK1/2) via phosphorylation of serine/threonine residues (Zong et al., 2013). Because ERK1/2 can be activated by a variety of stimuli, leading to cardiac remodeling, it may be an ideal target for improving cardiac remodeling (Zhong et al., 2015). GATA4 (GATA‐Binding Factor 4) is a member of the GATA family of zinc‐finger transcription factors (Arceci, King, Simon, Orkin, & Wilson, 1993). GATA4 is thought to regulate genes involved in embryogenesis and in cardiomyocytes differentiation and function. Studies have shown that GATA4 can be activated by phosphorylated‐ERK1/2, resulting in increased GATA4‐DNA binding capacity and activation of hypertrophic gene programs (T. Li, Liu, Hu, Ma, & Zhou, 2012; Zhong et al., 2015). One of the important findings of this study was that the activation of MEK1/2 and ERK1/2 was inhibited in the middle‐dose (25 mg/kg/day) and high‐dose (50 mg/kg/day) galangin treatment mice and 50 μM galangin compared with vehicle‐treated mice in response to pressure overload in vivo and H9c2 cells stimulated with Ang II in vitro.

Inflammation plays an important role in the development of AB‐induced cardiac remodeling (Han et al., 2017). Pressure overload or Ang II increases the release of proinflammatory cytokines IL‐1β, IL‐6 (Zhao, Ma, Guo, Li, & Liu, 2017). NF‐κB, as an important transcription factor, regulates many molecules involved in the early stages of immune response and the expressions of the proinflammatory cytokine, such as TNF‐α, IL‐1β, IL‐6, IL‐8, IL‐12, COX2, and so on (P. W. Liu et al., 2017). NF‐κB p65 is an important member of the NF‐κB family. Previous studies have shown that NF‐κB p65 is activated in the heart during the development of cardiac remodeling and promotes the production and release of inflammatory factors. Inhibition of NF‐κB P65 transcriptional activity ameliorates cardiac remodeling and suppresses the expressions of proinflammatory factors (Karuppagounder et al., 2016). IκBα (inhibitor of NF‐κB) is an inhibitor of NF‐κB. Under the resting state, IκBα and the NF‐κB p65 are inactivated in the cytoplasm. When the upstream signal activates and degrades IκBα, NF‐κB p65 is activated from inactivated state and transferred from cytoplasm to nucleus, binding to the corresponding inflammation‐related genes, initiating inflammatory cytokine transcription, and inducing inflammation (Tanaka & Iino, 2016). In our study, we showed that both AB surgery in vivo and Ang II stimulus in vitro increases the phosphorylation of IκBα□ and phosphorylation of NF‐κB p65. However, galangin administration reduces the heart expressions of phosphor‐IκBα□ and phospho‐NF‐κB p65 in vivo and in vitro.

Cardiomyocytes apoptosis increases when the heart is subjected to pressure overload or when cardiomyocytes are stimulated by Ang II (Yang et al., 2012; Yu, Hu, Li, Wang, & Chen, 2018). Galangin has been shown to modulate cell apoptosis (Tomar et al., 2017). In our experiments, we found that both AB surgery and Ang II‐stimulation increased the apoptosis ratio of H9c2 cells, increased the expression of apoptotic protein Bax, and decreased the expression of antiapoptotic protein Bcl2. However, galangin increased Bcl2 expression and decreased Bax expression in vivo and in vitro, as well as decreased the apoptosis ratio of H9c2 cells in vitro.

Myocardial fibrosis is another important pathological feature of pressure overload cardiac remodeling, which is characterized by the accumulation of collagen, increased extracellular matrix (ECM) deposition, impairs diastolic relaxation, and causes cardiac dysfunction, which is characterized by increased accumulation of collagen and deposition of ECM, decreased diastolic function and cardiac dysfunction. We observed the galangin reduced the cardiac fibrosis and inhibited collagen synthesis in vivo. To further discuss the molecular mechanism, we evaluated the role of galangin in TGF‐β/Smad signaling (a key role in the transcription of profibrotic genes). We have found that mice with galangin treatment attenuate the expression of TGF‐β1 and p‐smad2/t‐smad2 induced by pressure overload.

Taking the results of our study together, we showed the first evidence that galangin protects against cardiac hypertrophy, inflammation, apoptosis, and fibrosis was induced by pressure overload. Galangin also suppressed the activation of PI3K–Akt–GSK3β and MEK1/2–ERK1/2–GATA4 signaling in vitro and in vivo. Therefore, galangin may offer a promising effective approach for preventing cardiac remodeling.

## CONFLICT OF INTERESTS

The authors declare that there are no conflict of interests.

## AUTHOR CONTRIBUTIONS

H. B. W. and Q. Z. T. made substantial contributions to conception and design; H. B. W. and S. H. H. acquired data; M. X. and C. X. W. analyzed and interpreted data; H. H. L. and D. F. were involved in drafting the manuscript; J. Y., J. Y. and M. X. L. revised it critically for important intellectual content.

## Supporting information

Supplementary information

Supplementary information
