# Supplementary figures and images for: Galangin ameliorates cardiac remodeling via the MEK1/2–ERK1/2 and PI3K–AKT pathways
Source: J Cell Physiol. 2019 Feb 11;234(9):15654–67. doi: 10.1002/jcp.28216 (PMC6686163; doi:10.1002/jcp.28216)

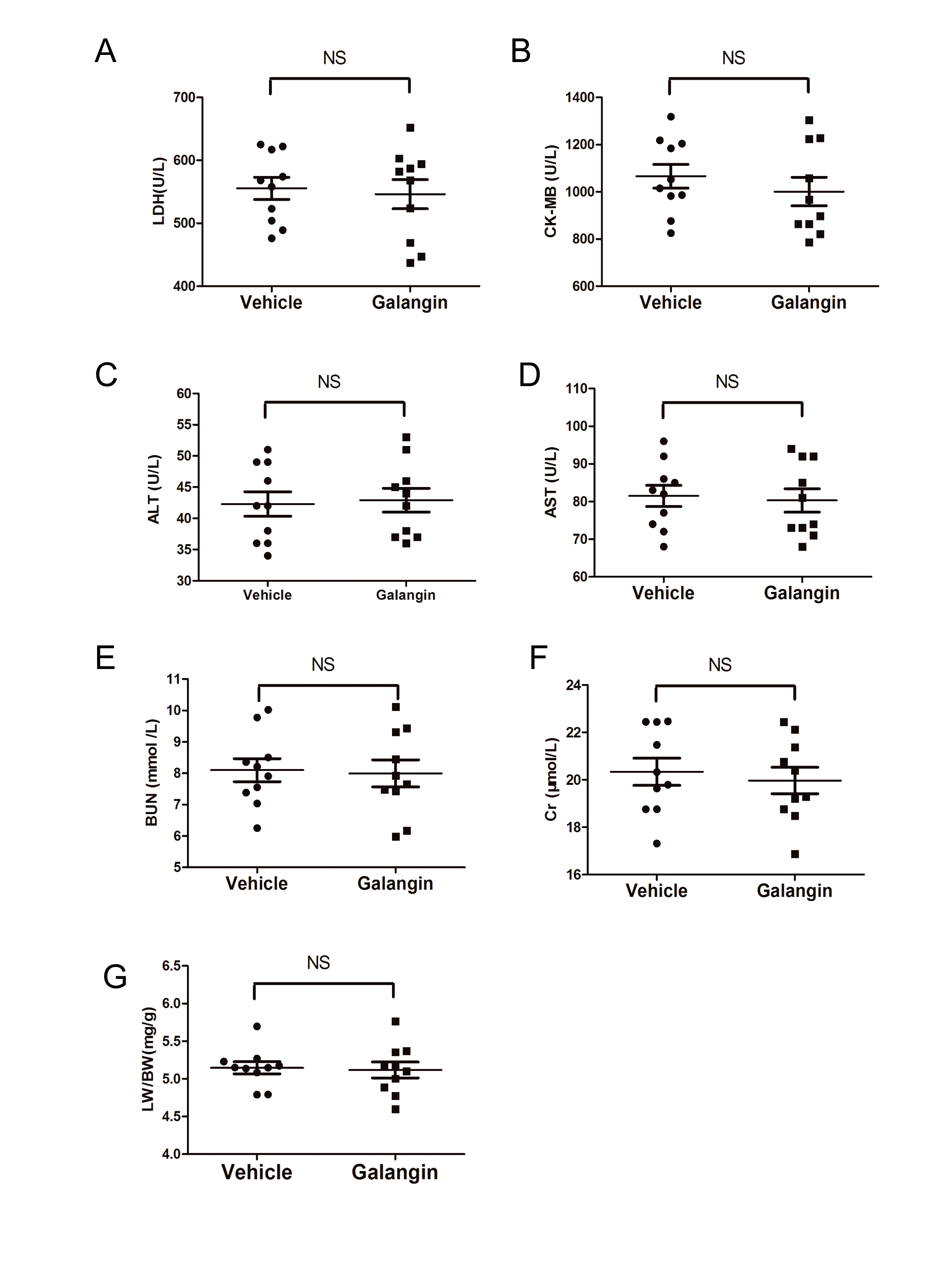

Supplement: Supplementary file 1 — Supplementary information [file JCP-234-15654-s001.tif]
