# Supplementary material for: Galangin ameliorates cardiac remodeling via the MEK1/2–ERK1/2 and PI3K–AKT pathways
Source: J Cell Physiol. 2019 Feb 11;234(9):15654–67. doi: 10.1002/jcp.28216 (PMC6686163; doi:10.1002/jcp.28216)
Supplement: Supplementary file 2 — Supplementary information [file JCP-234-15654-s002.docx]

**Supplementary Figure 1: Verify the toxicity and side effects of 50 mg/Kg/Day galangin on the heart, liver and kidney.** (A-B) Detection of serum myocardial enzymes LDH and CK-MB levels; (C-D) Detection of serum liver function indicators AST and ALT levels; (E-F) Detection of serum renal function indicators BUN and Cr levels; (G) Statistical results of LW/BW ratio. NS= no significance; LDH= lactate dehydrogenase; CK-MB= creatine kinase isoenzymes; AST=Glutamic oxaloacetic transaminase; ALT=Alanine aminotransferase; LW/BW= Lung weight/ body weight.
